# Biochemical studies on *Francisella tularensis* RelA in (p)ppGpp biosynthesis

**DOI:** 10.1042/BSR20150229

**Published:** 2015-11-26

**Authors:** Rachael C. Wilkinson, Laura E. Batten, Neil J. Wells, Petra C.F. Oyston, Peter L. Roach

**Affiliations:** * Department of Chemistry, University of Southampton, Southampton, SO17 1BJ, U.K.; † Institute for Life Sciences, University of Southampton, Southampton, SO17 1BJ, U.K.; § DSTL, Porton Down, Salisbury, SP4 OJQ, U.K.

**Keywords:** *Francisella*, kinetics, 5′, 3′-dibisphosphate guanosine (ppGpp), RelA (p)ppGpp synthetase I, stringent response, synthetase

## Abstract

The bacterial stringent response is induced by nutrient deprivation and is mediated by enzymes of the RSH (RelA/SpoT homologue; RelA, (p)ppGpp synthetase I; SpoT, (p)ppGpp synthetase II) superfamily that control concentrations of the ‘alarmones’ (p)ppGpp (guanosine penta- or tetra-phosphate). This regulatory pathway is present in the vast majority of pathogens and has been proposed as a potential anti-bacterial target. Current understanding of RelA-mediated responses is based on biochemical studies using *Escherichia coli* as a model. In comparison, the *Francisella tularensis* RelA sequence contains a truncated regulatory C-terminal region and an unusual synthetase motif (EXSD). Biochemical analysis of *F. tularensis* RelA showed the similarities and differences of this enzyme compared with the model RelA from *Escherichia coli*. Purification of the enzyme yielded a stable dimer capable of reaching concentrations of 10 mg/ml. In contrast with other enzymes from the RelA/SpoT homologue superfamily, activity assays with *F. tularensis* RelA demonstrate a high degree of specificity for GTP as a pyrophosphate acceptor, with no measurable turnover for GDP. Steady state kinetic analysis of *F. tularensis* RelA gave saturation activity curves that best fitted a sigmoidal function. This kinetic profile can result from allosteric regulation and further measurements with potential allosteric regulators demonstrated activation by ppGpp (5′,3′-dibisphosphate guanosine) with an EC_50_ of 60±1.9 μM. Activation of *F. tularensis* RelA by stalled ribosomal complexes formed with ribosomes purified from *E. coli* MRE600 was observed, but interestingly, significantly weaker activation with ribosomes isolated from *Francisella philomiragia*.

## Abstract (precis)

*Francisella tularensis* RelA shows significant sequence differences from other members of the RelA family of enzymes. In the present study, we describe the functional similarities and differences between *F. tularensis* RelA and the model RelA from *Escherichia coli*.

## INTRODUCTION

Bacteria rely on global metabolic regulation by the stringent response for survival as the environment becomes nutrient deficient [1,2]. This response is orchestrated by the synthesis and subsequent downstream effects of the signalling molecules guanosine penta- and tetra-phosphate [ collectively known as (p)ppGpp; 3]. In β- and γ-proteobacteria, synthesis of these signalling molecules is catalysed by the enzymes RelA and SpoT, members of the long RelA/SpoT homologue (RSH) superfamily [4]. Virtually ubiquitous across bacterial species, RSH proteins have been suggested as prospective novel anti-bacterial targets, with genetic knockouts showing attenuation in animal infection models [5–8].

**Figure 1:**
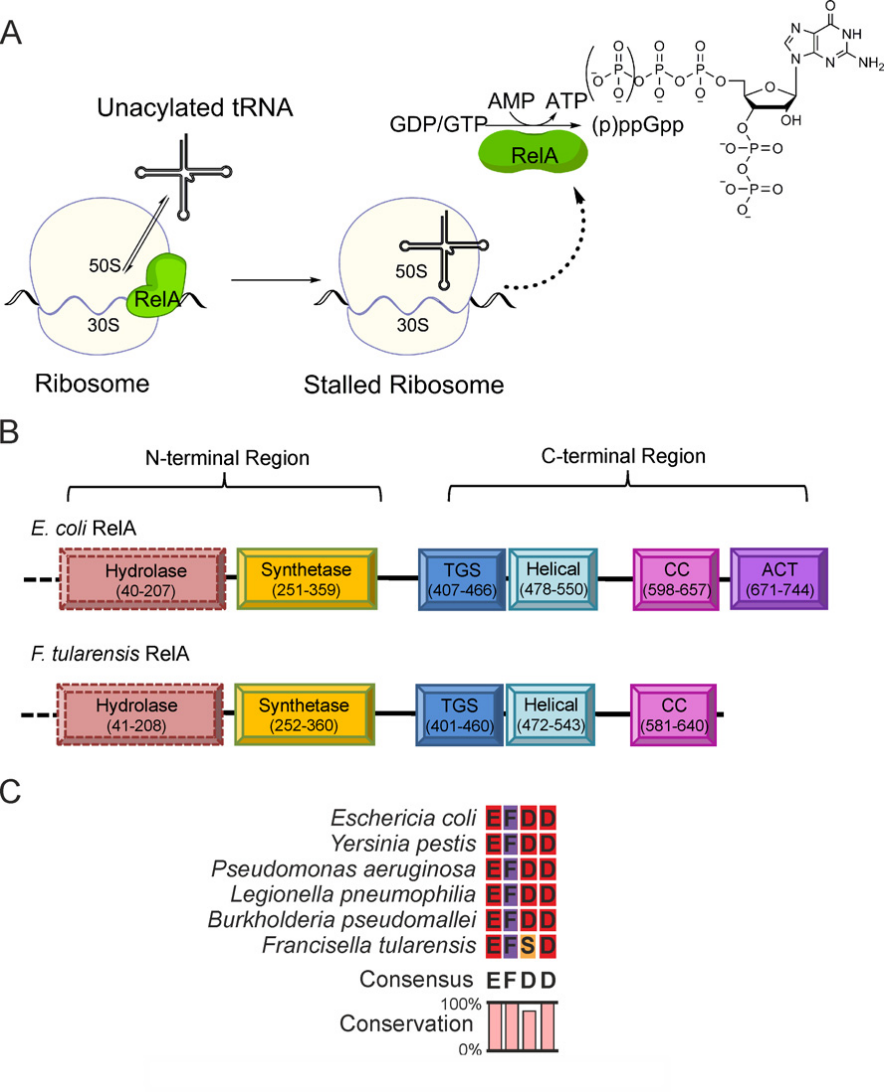
The extended hopping model for RelA activation, derived from the proposal of English et al. [15] Under conditions of amino acid starvation, unacylated-tRNA accumulates and binds to the acceptor (**A**) site of the ribosome, leading to stalling. This results in the release of RelA from the ribosome in an activated conformation, which catalyses the formation of (p)ppGpp. (**B**) Domain structure and corresponding amino acids for *Ec*RelA and *Ft*RelA. (**C**) Alignment of RelA enzymes synthetase domain active-site motif from pathogenic bacteria.

In *Escherichia coli*, and by inference other β- and γ-proteobacteria, the enzyme RelA is principally responsible for (p)ppGpp synthesis during amino acid starvation [9]. Since its discovery [10], the mechanism by which RelA activation leads to (p)ppGpp accumulation has been extensively studied [11–14]. A working hypothesis for RelA activation proposes that the enzyme is most active following its release from a stalled ribosomal complex [11,15] (Figure 1A). This hypothesis, termed the ‘extended hopping model’ [15], explains how the bacterium can respond to a reduced level of amino acids by sensing a hiatus in protein synthesis and is supported by both *in vitro* [11–13] and *in vivo* [15] experimental evidence.

The protein sequence for long RSH proteins [4] can be divided into an N-terminal region (containing catalytic sites) and a C-terminal region [containing regulatory threonyl-tRNA synthetase, GTPase and SpoT domain (TGS), conserved cysteine domain (CC), helical and aspartate kinase, chorismate mutase, TyrA domain (ACT) domains] connected by a linker region (Figure 1B) [4]. Long RSH proteins can be divided into bifunctional (Rel or SpoT) or mono-functional (RelA) enzymes. Both classes are capable of synthesizing (p)ppGpp but only bifunctional enzymes are capable of (p)ppGpp hydrolysis [16]. Studies with RelA fragments indicate the C-terminal region functions to regulate the catalytic synthetase domain [17] and contains the recognition features that permit homodimerization [18].

*F. tularensis* RelA (*Ft*RelA) contains a truncated C-terminal region, approximately 100 amino acids shorter than most RelAs and is one of only three RelA sequences known to not contain an ACT domain [4] (Figure 1B). Proteins containing ACT domains can be found throughout nature, displaying a conserved βαββαβ fold of the domain and are involved in small molecule ligand recognition [19,20]. This domain originally described as a ‘conserved, evolutionary mobile module’ is proposed to have evolutionarily fused with proteins to facilitate the regulation of their catalytic activity by the allosteric binding of small molecules [21].

Besides its truncated C-terminal region *Ft*RelA differs from the majority of other RelA enzymes in its synthetase active-site motif. The synthetase domain from RSH proteins are reported to contain either an RXKD or an EXDD motif, which are involved in the preferential binding of the pyrophosphate acceptor (i.e. GDP or GTP) [22]. In contrast, the synthetase domain in *Ft*RelA contains the alternative EXSD motif (Figure 1C), which interestingly can be found in a wide spectrum of *Francisella* species (Supplementary Figure S1). This alternative motif shows the replacement of the initial aspartate with a serine. The primary aspartate has been proposed to allow the co-ordination of a second magnesium ion [23]. The difference in this active-site motif in *Ft*RelA raised the question of whether the (p)ppGpp synthetase activity in the presence of an EXSD motif is more closely aligned to the RSH enzymes featuring RXKD or EXDD motifs.

The functional analysis of *Ft*RelA, described herein, offers insights into the importance of the distinctive sequence of the synthetase domain and the absence of the ACT domain, compared with other RelA enzymes.

## MATERIALS AND METHODS

DTT, BSA and antibiotics were purchased from Melford Laboratories; polyacrylamide-bis polyacrylamide [30% (w/v), 37:5:1], Bacto tryptone and yeast extract for culture media were purchased from Oxoid. Chelating fast flow resin and Superdex 200 resin were purchased from GE Healthcare; primers were purchased from Eurofins; restriction enzymes and *E. coli* strain K12 JM109 were purchased from New England Biolabs; pET16b plasmid was purchased from Merck Chemicals; *E. coli* RelA was expressed using a strain from the ASKA Clone library purchased from Shigen; *E. coli* MRE600 (C6) strain was purchased from NCTC. *Francisella philomiragia* was obtained from A.T.C.C.; mRNA was purchased from ATDBio. Unless stated otherwise all other reagents were purchased from Sigma–Aldrich or Fisher Scientific. Graphpad Prism version 6 for Windows was obtained from Graphpad Software.

### Cloning of *F. tularensis* RelA

The gene encoding *Ft*RelA, FTT_1508c, was amplified from *F. tularensis* subspecies *tularensis* SCHU S4 genomic DNA using a forward primer (5′- ccgccatgggtcatcatcatcatcatcatcaagttattgactctaaacttctagatagt) paired with a reverse primer (5′-cgcctcgagttagctgacctcttcattatcatc). The PCR product was digested with NcoI and XhoI and the resultant fragment was ligated into a backbone derived from NcoI/XhoI restricted pET16b. The sequence of the resultant plasmid pET16b::relA was verified by sequencing.

### Expression of *Ft*RelA

The plasmid pET16b::relA was chemically transformed into *E. coli* BL21 (DE3) pLysS competent cells (Sigma–Aldrich). Single colonies were used to inoculate 2YT media [24] (10 ml, containing 100 μg/ml ampicillin and 30 μg/ml chloramphenicol) and cultured overnight at 37°C. The overnight culture was used as a 1% inoculum for flasks of 2YT (4×1.25 litre) which were induced with IPTG (final concentration of 0.4 mM) when the absorbance at 600 nm (*A*_600_) reached 0.6 and then cultured overnight at 16°C. The cell pellet was then collected by centrifugation (average yield of 5 g/ 1 litre of culture) and stored at–80°C.

### Purification of *Ft*RelA

Frozen cell pellet (typically 15 g) was resuspended (3 × v/w cell pellet) in buffer A [50 mM Tris, pH 8.0, 500 mM NaCl, 3 mM β-mercaptoethanol, 15% (v/v) glycerol and 20 mM imidazole]. Lysozyme (5–15 mg) and a Roche protease inhibitor tablet (1 per 50 ml) were added to the cell suspension and left to stir (4°C, 30 min). Cells were lysed by sonication (4°C, 20×30 s with 30 s rest) and cellular debris removed by centrifugation (23446 ***g***, 4°C, 30 min). The resultant cleared lysate was applied (4 ml·min^−1^) to a Ni-IDA (iminodiacetic acid) Sepharose Fast Flow Column (50 ml bed volume). The column was then washed (4 ml·min^−1^) with buffer A until the absorption of the eluate at 280 nm (*A*_280_) returned to baseline. Elution (4 ml·min^−1^) of *Ft*RelA was achieved using a gradient of imidazole from 20 to 500 mM (buffer B, as buffer A but with 500 mM imidazole) over 5 column volumes. Fractions containing *Ft*RelA were pooled and dialysed against buffer C [2×1 litre, 50 mM Tris, pH 8.0, 300 mM NaCl, 1 mM DTT, 15% (v/v) glycerol] and concentrated to 10–15 mg/ml in an Amicon Pressure Cell (30 kDa PES filter, Sartorius) before overnight storage at 4°C. The concentrated *Ft*RelA (3 ml) was then applied (2 ml·min^−1^) to a gel filtration column (HiLoad 26/60, Superdex 200, prep grade) pre-equilibrated in buffer C. The purest fractions of *Ft*RelA, as judged by SDS/PAGE, were pooled and concentrated (as described above) to ∼4 mg/ml (∼50 μM), then aliquoted (typically 0.2 ml) and stored at–80°C. For biochemical experiments, *Ft*RelA aliquots were defrosted and used only once. Each batch of *Ft*RelA was used within 8 weeks of freezing.

### Characterization of *Ft*RelA multimeric state by gel filtration

A Superdex 200 column (10 mm × 300 mm) was used to estimate the apparent molecular mass of purified *Ft*RelA (∼1 mg/ml, ∼13.5 μM). Protein samples were applied (1 ml·min^−1^) to a pre-equilibrated column in buffer D (50 mM Tris/HCl, pH 7.5, 100 mM KCl). Cytochrome C, carbonic anhydrase, BSA, alcohol dehydrogenase and β-amylase (Sigma–Aldrich) were used as protein standards for calibration of the column. Elution of the protein samples were monitored by absorbance at 280 nm.

### HPLC analysis of *Ft*RelA activity

*Ft*RelA activity assays, substrate specificity assays and assays to identify and quantify activating factors were all analysed by IP RP (ion pair reverse phase) HPLC, using methods adapted from Cordell et al. [25]. Injected samples (40 μl) were chromatographed on a reverse phase column [Gemini C18, 150×4.6 mm^2^ 5 micron (Phenomenex)] at a flow rate of 0.8 ml·min^−1^ with UV detection at 260 nm. The mobile aqueous phase was 95% water with 5% methanol and organic phase was 20% water with 80% methanol. Both phases contained DMHA (*N*,*N*-dimethylhexylamine; 15 mM) and were adjusted to pH 7.0 with acetic acid. Nts were eluted with the following gradient: 0–5 min, isocratic, 25% organic buffer; 5–27 min, gradient 25%–60%; 27–28 min, gradient 60%–100%; 28–33 min, isocratic, 100%; 33–34 min, gradient 100%-25%; 34–44 min, isocratic 25%.

### Substrate specificity of *Ft*RelA

Reaction mixtures (100 μl) were prepared with components at the following final concentrations: ribonucleotide di- or triphosphates (2 mM, either G, T or C), ATP (2 mM) and *Ft*RelA (10 μM) in assay buffer (20 mM Tris, pH 8.0, 15 mM KCl, 15 mM MgCl_2_, 1 mM β-mercaptoethanol). Reactions were incubated at 30°C for 60 min, prior to quenching by heating. All reactions quenched by heat were maintained at 80°C for 2 min. The reaction mixtures were then kept on ice to ensure full protein precipitation (typically 10 min), before clearing by centrifugation (13684 ***g***, 4°C, 5 min). An aliquot (40 μl) of the supernatant was then analysed by IP RP HPLC. Assays to determine substrate selectivity under modified conditions are described in ‘Supplementary Methods’ 1 and 2.

### Steady state kinetics for *Ft*RelA and substrates

Reaction mixtures for time course experiments (1 ml) contained *Ft*RelA (10 μM) and were made up in assay buffer. When saturating, nts (GTP or ATP) were included at 2 mM. Reactions were incubated at 30°C and at selected time points, aliquots (100 μl) were withdrawn and reaction quenched by heating. Precipitated protein was removed as described above and a sample (40 μl) was analysed by IP RP HPLC. The concentrations of nts were quantified by comparison with a standard calibration curve. The formation of product nts over time (never more than 15% substrate turnover) was fitted to a linear function to determine initial rates (Supplementary Table S1). Initial rates, *v*, were fitted to an allosteric sigmoidal function (eqn 1) using Graphpad Prism software where *V*_max_ is the rate of reaction at substrate saturation, *K*_1/2_ is the concentration of substrate giving a rate of half of *V*_max_ and h is the apparent Hill constant.

1 $$v=\frac{V_{max}S^{h}}{\left(K_{{}^{1}{/}_{2}}^{h}+S^{h}\right)}$$

### [^31^P]-NMR analysis of *Ft*RelA activity

*Ft*RelA-catalysed reaction time courses were monitored with [^31^P]-NMR. All data were recorded on a Bruker AVII400 FT-NMR Spectrometer using a 10-mm auto-tune and match broadband probe tuned to the sample prior to data collection (data acquisition size=65536 points; sweep width=395 ppm; 512 scans using 90° pulse with a total acquisition time of 2.5 s per scan; chemical shifts referenced to H_3_PO_4_). Reaction mixtures (3 ml) were prepared in assay buffer and contained GTP (2 mM), ATP (2 mM) and 10% ^2^H_2_O. The reaction was initiated through the addition of *Ft*RelA (10 μM), thoroughly mixed and then data collected at 25°C for 22 min followed by 22 min bins for the duration of the experiment (typically 110 min). Peak integrals were calibrated against a spectrum recorded prior to initiation. At each substrate concentration, the formation of product nts over time was fitted to a linear function to determine the rate of product formation. These rates were then fitted to an allosteric sigmoidal curve (eqn 1).

### Expression and purification of ribosomes

Ribosomes were isolated from both *F. philomiragia* and *E. coli* MRE600. For the purification of ribosomes from *E. coli*, overnight cultures of *E. coli* MRE600 (LB media, 10 ml) were used to inoculate flasks of LB media (1 litre) supplemented with MgSO_4_ (10 mM). Cultures were grown at 37°C to an *A*_600_ of 0.4; cells were then pelleted by centrifugation (3501 ***g***, 4°C, 30 min). For the purification of ribosomes from *F. philomiragia*, overnight cultures of *F. philomiragia* [trypticase soy broth (TSB), 10 ml] were used to inoculate flasks of TSB (1 litre) supplemented with L-cysteine (1 g/l). Culture was grown at 37°C to an *A*_600_ of 1.0 and the cells collected by centrifugation as described above. Cell pellets were washed and stored as described by Maguire et al. [26]. SulfoLink-cysteine resin was prepared and ribosomes purified as described for *E. coli* ribosomes by Maguire et al. [26]. Fractions (10 ml) containing ribosomes, as assessed by absorption at 260 nm, SDS/PAGE and measurement of protein content by the method of Bradford [27], were pooled and concentrated to 4.96 and 8.93 μM for *F. philomiragia* and *E. coli* respectively in an Amicon Pressure Cell (30 kDa PES filter, Sartorius). Concentrated ribosomes were aliquoted (200 μL) and stored at–80°C. RNA was isolated from ribosomal samples using the GeneJet RNA purification kit (Thermo Scientific) and analysed by bleach RNA gel electrophoresis as described by Aranda et al. [28].

### Ribosome-activated *Ft*RelA activity assays

Reaction mixtures containing stalled ribosomal complexes were prepared in assay buffer with purified ribosomes (0.2 μM), purified RelA (0.5 μM), ATP (2 mM) and each RNA species (0.3 μM). RNA species include: mRNA (5′-caaggagguaaaaauggucgucgcacgu) [12], tRNA^fmet^ (from *E. coli* MRE600) and tRNA^val^ (from *E. coli* MRE600). Reaction mixtures were incubated at 30°C for 5 min prior to initiation by the addition of GTP (2 mM final concentration). Reaction mixtures were incubated for 60 min at 30°C prior to quenching by heating. Precipitated protein was removed as described above and a sample (40 μl) was analysed by IP RP HPLC.

### Product activation of *Ft*RelA

The nt ppGpp (5′,3′-dibisphosphate guanosine) was synthesized and purified as described by Carmona et al. [29] with minor modifications (Supplementary Method S3). Reaction mixtures (100 μl) were prepared in assay buffer with components at the following final concentrations: ribonucleotide triphosphates (2 mM, both A and G), *Ft*RelA (5 μM) and purified ppGpp, AMP or KH_2_PO_4_ (at indicated concentrations). Reactions were incubated at 30°C for 60 min prior to quenching by heating. Protein was precipitated and removed from samples as detailed above, prior to analysis of the sample by IP RP HPLC. Assays were prepared alongside a negative control containing no additional small molecular component. The EC_50_ value was calculated with a sigmoidal dose-response (variable slope) curve (eqn 2), using Graphpad Prism software where *Y* is the rate, *Y*_max_ is the maximum rate, *Y*_min_ is the basal rate and EC_50_ is the concentration of ligand required to give 50% of full activation and h is the apparent Hill slope.

2 $$Y\;=Y_{\min}\;+\;\frac{\left(Y_{\max}-Y_{\min}\right)}{1\;+10^{\left(\mathrm{LogE}C_{50}\;-\;X\right)\;-\;h}}$$

## RESULTS

### Expression and purification of *Ft*RelA

**Figure 2:**
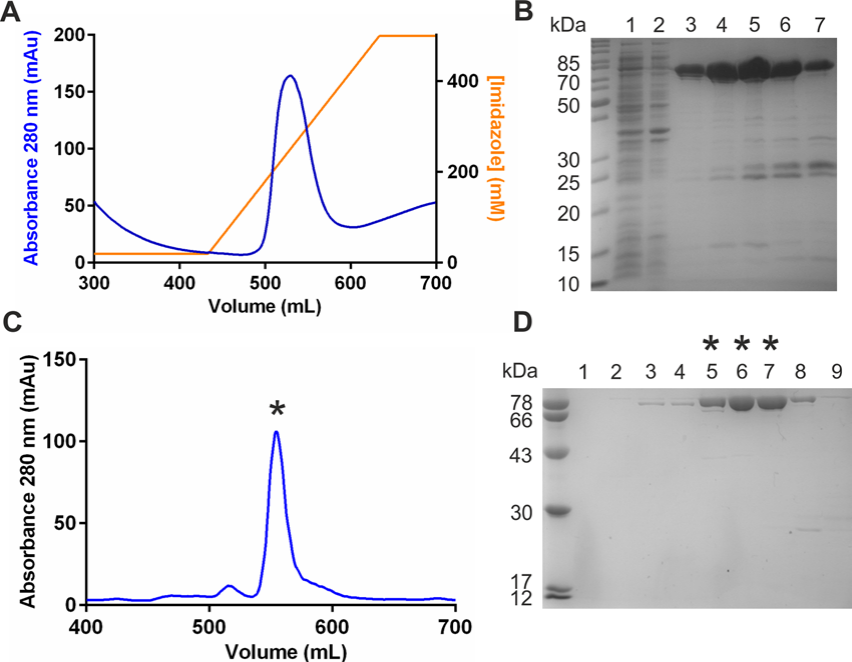
The purification of *Ft*RelA by sequential Ni-IDA and size exclusion chromatography steps (**A**) Absorption trace (280 nm) for the Ni-IDA purification of *Ft*RelA. (**B**) SDS/PAGE analysis of *Ft*RelA Ni-IDA purification. Lanes: 1, cleared lysate; 2, flow through; 3–7, eluate fractions from Ni-IDA chromatography. (**C**) Absorption trace (280 nm) for the size exclusion (Superdex 200) chromatography purification of *Ft*RelA. (**D**) SDS/PAGE analysis of *Ft*RelA purification. Lanes: 1–8, eluate fractions from size exclusion chromatography; those marked with * correspond to the peak similarly marked in (**C**).

The RelA encoding gene, FTT_1508c, from *F. tularensis* subspecies *tularensis* SCHU S4 was cloned with primers designed to encode an N-terminal His_6_-tag to facilitate subsequent protein purification. Optimized expression of *Ft*RelA in *E. coli* BL21 (DE3) pLysS was achieved by maintaining the cultures at 16°C overnight after induction. The *Ft*RelA purification required two chromatographic steps with the initial purification using Ni-IDA affinity chromatography (Figure 2A). SDS/PAGE analysis showed a distinct band corresponding to the expected molecular mass (74 kDa) of *Ft*RelA alongside several minor impurities (Figure 2B). *Ft*RelA was further purified in a polishing step, using size exclusion chromatography (Superdex 200, Figures 2C and 2D). The resultant highly purified *Ft*RelA was isolated in a yield of 12 mg/ g cell pellet and could be concentrated up to 10 mg/ml (∼100 μM).

### Multimeric state of purified *Ft*RelA

**Figure 3:**
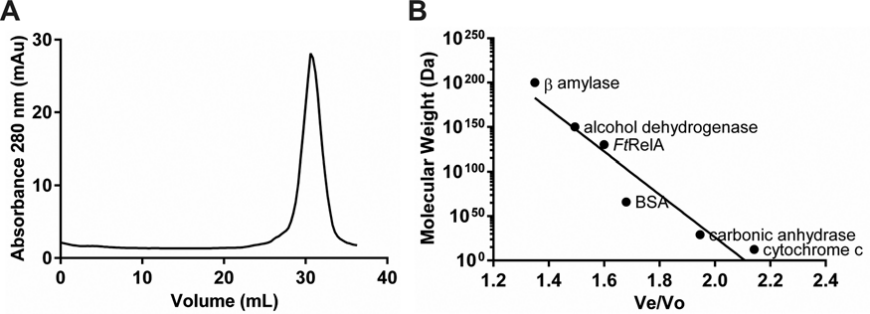
Analysing the multimeric state of purified *Ft*RelA (**A**) Absorption trace (280 nm) for size exclusion chromatography (Superdex 200) yielding a single peak with an apparent molecular mass of 128±1.57 kDa corresponding to the dimeric state of the enzyme (calculated molecular mass of dimer 148 kDa). (**B**) Calibration curve for apparent molecular mass determination.

The multimeric state of purified *Ft*RelA was determined using size exclusion chromatography and comparison with protein standards. *Ft*RelA was shown to form one distinct peak which had an apparent mass of 128±1.57 kDa that approximately corresponds to a dimer state (calculated molecular mass of 148 kDa; Figure 3).

### Substrate specificity of *Ft*RelA

**Figure 4:**
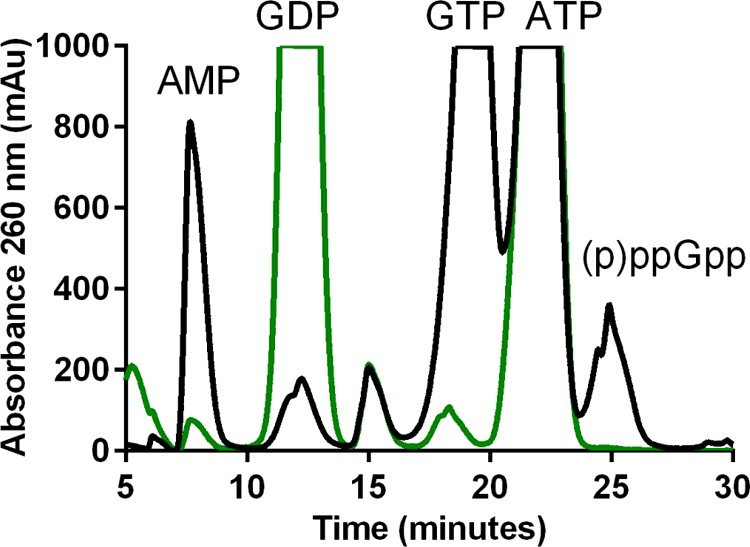
Substrate specificity of *Ft*RelA *Ft*RelA activity assays, with either GTP (solid black line) or GDP (dashed green line) as the pyrophosphate acceptor, were stopped after 1 h and analysed by IP RP-HPLC.

As other RSH enzymes have been shown to utilize GDP and GTP as pyrophosphate acceptors [30–33], the specificity of *Ft*RelA for these nts was investigated. End-point *Ft*RelA activity assays were prepared with *Ft*RelA, ATP and either GDP, GTP or, as a negative control CTP (Supplementary Figure S2). Efficient formation of AMP and the 3′-pyrophosphorylated product were only observed in the presence of GTP as a co-substrate (Figure 4; Supplementary Figure S2); GDP and CTP were not accepted as substrates. Very low concentrations of AMP are still measurable for activity assays with GDP as a pyrophosphate acceptor (Figure 4), but the formation of ppGpp is not observed. *Ft*RelA remained selective for GTP as a pyrophosphate acceptor in the presence of stalled ribosomal complexes (Supplementary Figure 3A) but accepted GDP as a substrate in the presence of methanol (30% v/v, Supplementary Figure 3B)

### Kinetic characterization of *Ft*RelA basal activity analysed by IP RP HPLC

**Figure 5:**
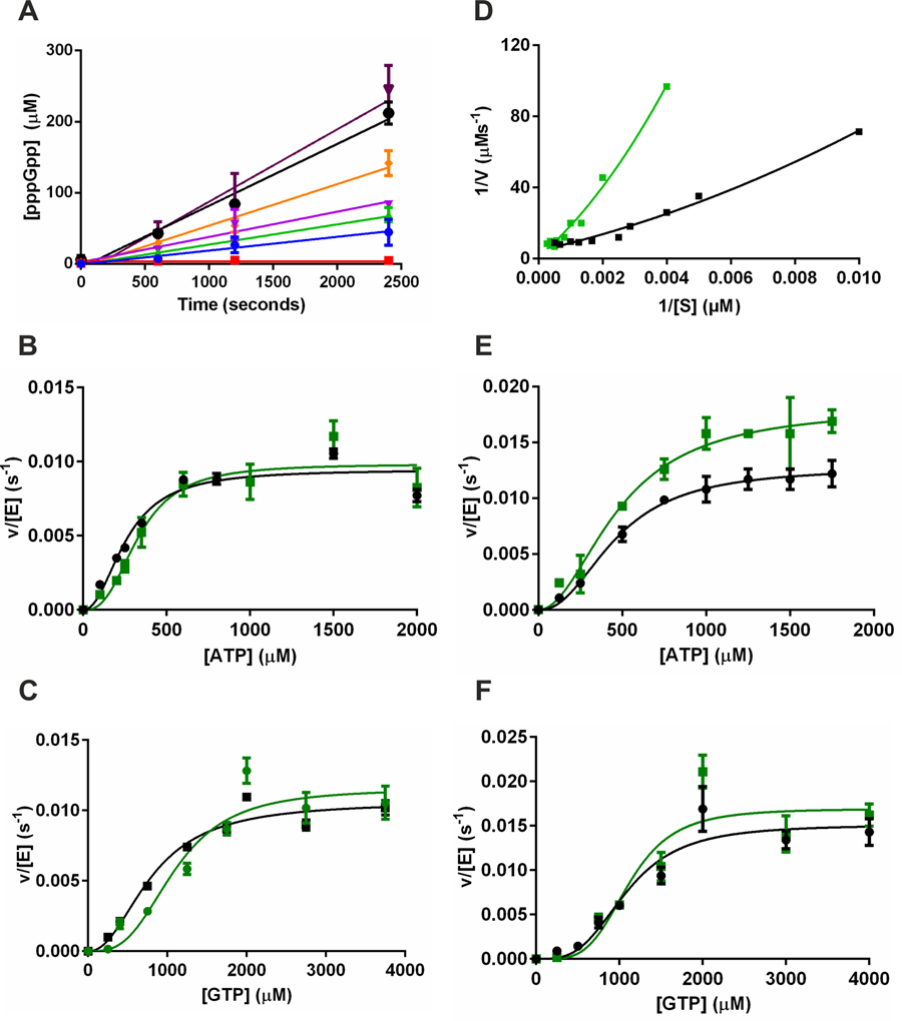
Kinetic analysis of *Ft*RelA Activity (**A**) Time courses of AMP formation at varying GTP concentrations in *Ft*RelA activity assays determined by HPLC analysis; 0.25 mM (red), 0.4 mM (blue), 0.75 mM (green), 1 mM (lilac), 1.25 mM (orange), 1.75 mM (black), 2.75 mM (violet). (**B**) Saturation activity curves for ATP substrate, measured using HPLC analysis. (**C**) Saturation activity curves for GTP substrate, measured by HPLC analysis. (**D**) Lineweaver–Burk plot for *Ft*RelA calculated for ATP (black line) and GTP (green line) when analysed by IP RP HPLC. (**E**) Saturation activity curves for ATP substrate, measured by [^31^P]-NMR analysis. (**B**) Saturation activity curves for GTP substrate, measured by [^31^P]-NMR analysis. All saturations curves using rates derived from AMP formation are denoted with a black line and those derived from (p)ppGpp formation are denoted with a green line.

To obtain steady state kinetic parameters for *Ft*RelA, a series of reaction time course experiments over a range of substrate concentrations were prepared. For time course experiments, initial rates resulting in less than 15% turnover of substrate were determined from HPLC quantification of products in aliquots withdrawn at 0, 10, 20 and 40 min (Figure 5A; Supplementary Table S1). The HPLC analysis provided measurements of the products concentrations: the AMP peak was clearly resolved, however slow degradation of pppGpp (5′-triphosphate-3′-diphosphate guanosine) to ppGpp resulted in two partially overlapping peaks, which were combined to give an overall (p)ppGpp concentration (Supplementary Figure S4). A better fit of the velocity curves (plotting rate against concentration of substrate) was achieved for a sigmoidal (*R*^2^=0.947) rather than a hyperbolic function (*R*^2^=0.899) for AMP production (Figures 5B and 5C; Table 1). The Lineweaver–Burk plot for *Ft*RelA steady state data was observed to curve in a manner consistent with positive co-operativity [34] (Figure 5D). Fitting to eqn. (1) yielded the kinetic parameters in Table 1 including *K*_1/2_ which denotes the concentration of substrate at which half the maximal activity of the enzyme is achieved. The *K*_1/2_ values for ATP as a substrate are within error when calculated from rate of formation of AMP or (p)ppGpp, with values of 259±37.2 μM and 332±47.8 μM respectively. The *K*_1/2_ values for GTP as a substrate when calculated from AMP or pppGpp formation are 800.7±115.6 μM and 1095±183.8 μM respectively, which are also within error.

**Table 1:** Kinetic parameters derived from HPLC analysis of *Ft*RelA activity assays for GTP and ATP substrates. *R*^2^ is a measure of goodness of fit The apparent Hill constant, h, is defined in equation 1 (above).

Substrate	ATP		GTP	
Product measured	AMP	(p)ppGpp	AMP	(p)ppGpp
*V*_max_/× 10^−3^ s^−1^	9.46±0.77	9.83±0.82	10.59±0.89	11.54±1.5
*K*_1/2_/*μ*M	259±37.2	332±47.8	800.7±115.6	1095±183.8
h	2.17±0.7	2.71±0.96	2.18±0.53	3.07±1.29
*R*^2^	0.95	0.94	0.97	0.93

### Kinetic characterization of *Ft*RelA basal activity by [^31^P]-NMR

As the sigmoidal saturation kinetic profile of *Ft*RelA observed by HPLC analysis is somewhat unusual, we sought to verify it using [^31^P]-NMR as an alternative measurement technique. Despite NMR not often being suitable for monitoring enzymatic reactions, due to its intrinsic low sensitivity and resultant long acquisition times, the relatively slow turnover of *Ft*RelA in the absence of activators (as judged by HPLC analysis) suggested [^31^P]-NMR might also provide a useful approach for this enzyme. Using nt standards dissolved in enzyme assay buffer, the signals for each phosphorus atom in the substrates and products were identified (Supplementary Table S2). In preliminary NMR experiments, some of these signals were observed to overlap including those for the 5′-α and 3′-α of ppGpp (∼6 ppm). The signal for the α-phosphate of AMP (3.32 ppm; Supplementary Figure S5) however was well resolved. Despite the formation of an individual peak for the 5′-β phosphate of pppGpp (–19.88 ppm; Supplementary Figure S5), the observed overlap with the neighbouring peak (5′-β phosphate of GTP/ATP) and slow hydrolytic conversion of pppGpp to ppGpp resulted in an increased error in measuring pppGpp formation. This resulted in the higher derived rates and apparent *V*_max_ determined from calculated initial rates of pppGpp formation (Figure 5).

The conversion of GTP to pppGpp was shown to occur in a highly specific manner, with only one minor by-product being observed, inorganic phosphate (1.94 ppm; Supplementary Figure S5), which accumulates during the activity assays. This observation is consistent with either the instability of pppGpp under assay conditions or the presence of a very low level of contaminating phosphatase activity [35].

The intrinsic insensitivity of [^31^P]-NMR results in a relatively weak signal, but 22-min spaced time points partly compensated for this. However, the resultant data come with a caveat that a significant proportion of the substrates (∼30%) had been turned over by the third time point (66 min). Somewhat surprisingly, these [^31^P]-NMR time courses (Supplementary Figure S6) showed approximate linearity in product formation to at least 1 h and reaction rates were calculated within this linear range (Supplementary Figure S7; Supplementary Table S3). The rates determined at a selected range of substrate concentrations were plotted to yield velocity curves (Figures 5E and 5F). Once again, these fitted to a sigmoidal function comparable to the results observed with IP RP HPLC analysis of *Ft*RelA activity assays, including the *K*_1/2_ and *V*_max_ for GTP and ATP (Supplementary Table S4). Data obtained by [^31^P]-NMR should however be considered as an estimate only, due to the higher than normal proportion of substrate turnover measured. Kinetic data sets measured by [^31^P]-NMR and HPLC were fitted to eqn (1) with *V*_max_ and *K*_1/2_ as global (shared) constants to derive overall kinetic parameters (Table 2; Supplementary Figure S8). For the reasons previously discussed, the initial rates calculated by [^31^P]-NMR analysis of pppGpp formation were omitted from this global fit.

### Activation of *Ft*RelA by small molecules

**Figure 6:**
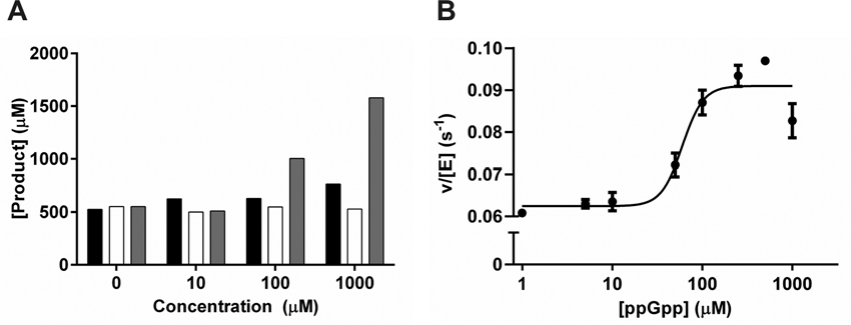
Activation of *Ft*RelA by potential small-molecule activators (**A**) Graph shows enzyme activity when incubated with low (10 μM), medium (100 μM) and high concentrations (1000 μM) of AMP (black), inorganic phosphate (white) or ppGpp (grey) in addition to substrates. (**B**) Dose-response (variable slope) curve for activation of *Ft*RelA by ppGpp.

Shyp et al. [14] have described the positive regulation of *E. coli* RelA (*Ec*RelA) by the product of GDP pyrophosphorylation, ppGpp [14]. Kinetic analysis using both HPLC and [^31^P]-NMR methods showed positive co-operativity of *Ft*RelA activity and it was of interest to determine if small molecules such as ppGpp or other small ligands were regulating activity. The effect of adding putative activating factors, AMP, phosphate or ppGpp prior to initiating the activity assay at low (10 μM), medium (100 μM) or high (1000 μM) concentrations on the rate of product formation was measured using IP RP HPLC analysis. The background concentration of additional ppGpp or AMP was subtracted to give the concentration of each product newly formed during the experiment. No significant activation was observed in the presence of additional inorganic phosphate (KH_2_PO_4_) or AMP (Figure 6A); however, in the presence of medium to high concentrations of ppGpp, activation was observed. Repeating this activation measurement over a wider range of ppGpp concentrations (0–1000 μM) allowed determination of an EC_50_ for ppGpp of 60±1.9 μM (Figure 6B) and a maximal 1.5-fold activation, similar to the approximate 2-fold activation observed for *Ec*RelA [14].

**Table 2:** Calculated kinetic parameters for *Ft*RelA when a global fit is applied to both [^31^P]-NMR (AMP) and IP RP HPLC (AMP and pppGpp) data sets. *R*^2^ is a measure of goodness of fit. The apparent Hill constant, h, is defined in equation 1 (above).

Substrate	ATP	GTP
*V*_max_/× 10^−3^ s^−1^	10.79±0.59	12.6±1.19
*K*_1/2_/*μ*M	344.3±33.95	1026±134.5
h	2.26±0.44	2.58±0.70
*R*^2^	0.932	0.881

### Activation of *Ft*RelA by stalled ribosomal complexes

**Figure 7:**
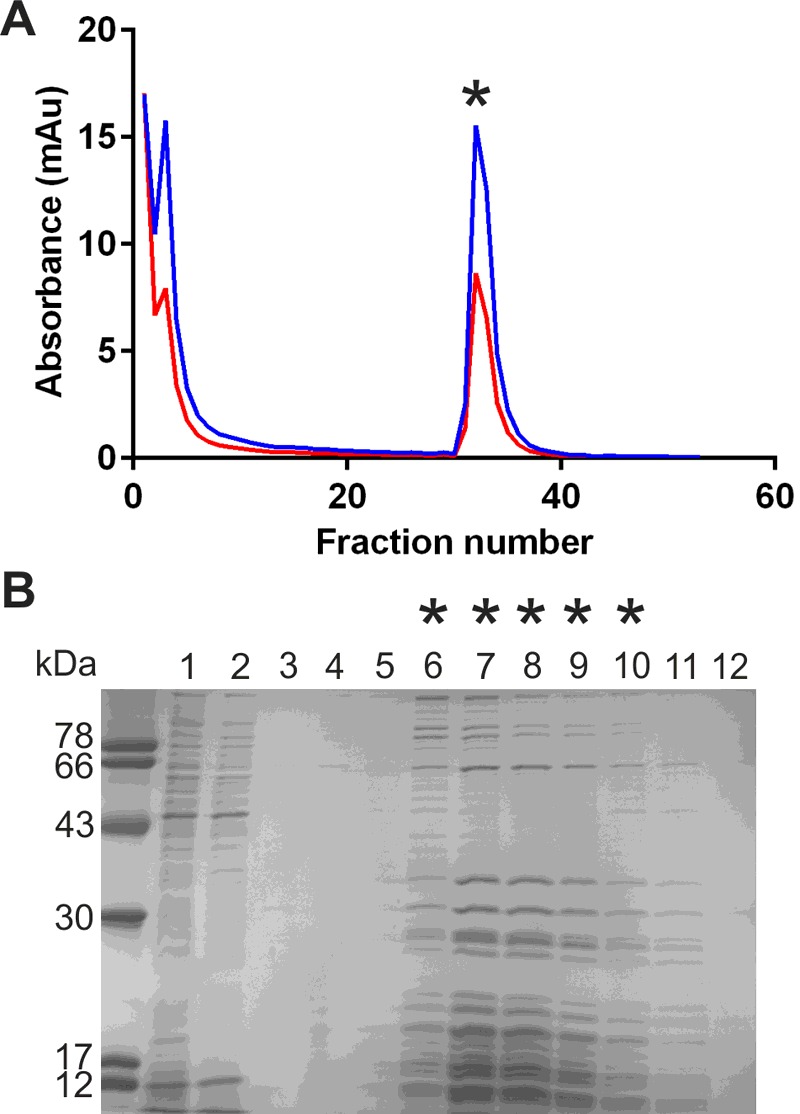
Purification of *F. philomiragia* ribosomes by SulfoLink-cysteine chromatography (**A**) Absorption traces at 260 nm (blue trace) and 280 nm (red trace) for fractions collected during purification. (**B**) SDS/PAGE analysis of *F. philomiragia* ribosome purification. Lanes: 1, cleared lysate; 2, flow through; 3–12, eluate fractions from SulfoLink-cysteine chromatography; those marked with * relate to the peak similarly marked in (**A**).

Bio-safety considerations encouraged us to explore alternatives to *F. tularensis* as sources for ribosome isolation. Two alternative ribosome sources were investigated. Firstly, the well characterized *E. coli* MRE600 strain which lacks ribonuclease I [36] and has consequently been widely used for ribosome purification [26,37,38]. Secondly, another member of the *Francisella* genus, *F. philomiragia*, which is of low virulence [39] and also encodes within its genome a RelA enzyme lacking the ACT domain in its C-terminus [4]. Maguire et al. [26] developed an affinity chromatography method for ribosome purification using cysteine coupled SulfoLink resin (Pierce). Previous purification of ribosomes from clinical isolates of pathogenic bacteria using this method [26], suggested its potential use for the isolation of ribosomes from *F. philomiragia*. Ribosomes were isolated from bacterial cells collected from early-mid logarithmic phase cultures, as established by growth curves (Supplementary Figure S9), to ensure the optimal recovery of ribosomes. The protein and RNA content of purified ribosomes were analysed primarily by SDS/PAGE and absorbance traces at 260 and 280 nm (Figure 7; Supplementary Figure S10). Purification of ribosomes by this method can contain tRNA, which is thought to also interact with the resin [40]. Contaminating tRNA was removed from ribosomal preparations by ultrafiltration. Purified *E. coli* MRE600 ribosomes were shown to contain all ribosomal RNA species by bleach agarose gel electrophoresis (Supplementary Figure S10 B); however, purified RNA from *F. philomiragia* proved too unstable for analysis by this method.

**Figure 8:**
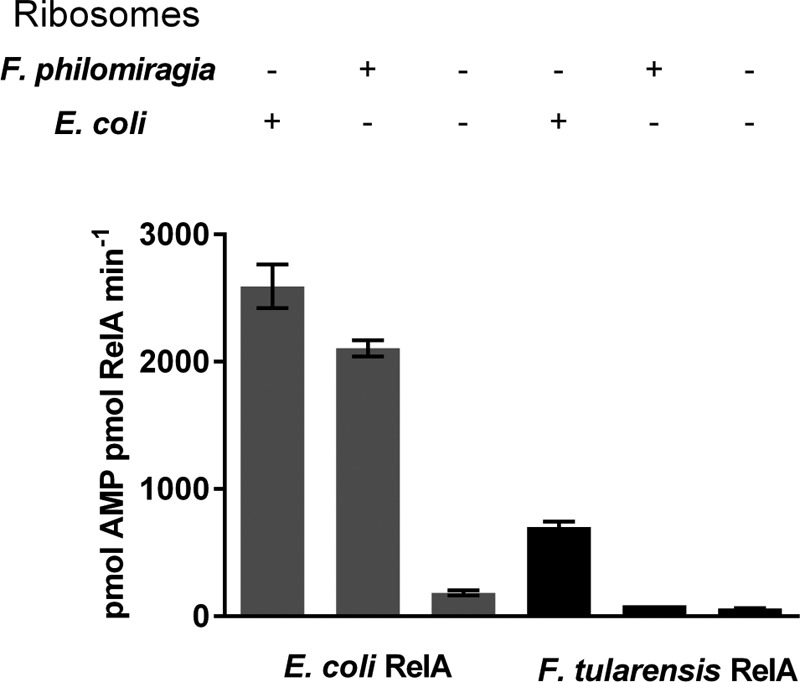
Activation of RelA by stalled ribosomal complexes Activation of *Ft*RelA and *Ec*RelA by stalled ribosomal complexes comprises either *F. philomiragia* ribosomes or *E. coli* MRE600 ribosomes, compared with basal RelA activity.

End-point (1 h) activity assays for both *Ec*RelA and *Ft*RelA were prepared containing one of the following: *F. philomiragia* ribosomes, *E. coli* ribosomes or in the absence of any ribosomes. Rates are shown in units of picomoles AMP per picomoles RelA per minute (Figure 8) to allow comparison with previously published data for activated *Ec*RelA [41]. Both *Ec*RelA and *Ft*RelA demonstrated basal levels of activity in the absence of stalled ribosomes, as has been noted previously for *Ec*RelA and Rel_Mtb_ [42,43]. *Ft*RelA displayed strong activation by *E. coli* ribosomes, with an 11-fold increase in activity compared with the basal level (Figure 8). We were unable however to show strong activation of *Ft*RelA in the presence of *F. philomiragia* ribosomes, with only a modest 1.39-fold increase observed (Figure 8). Conversely *Ec*RelA showed strong activation in the presence of either *F. philomiragia*, with an 11-fold increase or *E. coli* ribosomes with a 16-fold increase, respectively (Figure 8). Maximal *Ft*RelA activity (701.5±30.5 pmol AMP per pmol RelA per min) did not reach that of *Ec*RelA (2952±99.14 picomoles AMP per picomoles RelA per minute) under any conditions tested.

## DISCUSSION

Virtually ubiquitous across bacterial species, the stringent response is co-ordinated by signalling molecules (p)ppGpp and is important in bacterial survival under nutrient deficient conditions. In β- and γ-proteobacteria, the principle enzyme responsible for (p)ppGpp synthesis is RelA [4] and yet research on this enzyme has focused almost exclusively on *E. coli* RelA as a model enzyme [11–13,30]. In the present study, we discuss the relevance of the distinctive synthetase active-site motif and C-terminal truncation within *Ft*RelA in the functional similarities and differences when compared with other defined RelA enzymes.

Purification of this enzyme by nickel affinity chromatography followed by size exclusion chromatography yielded highly purified RelA, which could be concentrated to a 10-fold higher concentration than that reported for *E. coli* RelA [13]. The higher solubility of *Ft*RelA may be of practical value in future crystallization studies.

Previous work with *E. coli* RelA has demonstrated the enzyme's ability to dimerize via its C-terminal region [17,18]. The ability of *Ft*RelA to therefore dimerize was of interest given its truncated C-terminus. In the present study however, we demonstrate that *Ft*RelA forms a stable dimer upon purification. We postulate that the presence of the identified key residues for *Ec*RelA dimerization Cys^612^, Asp^637^ and Cys^638^ [17] in *Ft*RelA facilitate the formation the dimer without the requirement of downstream residues. We note that this may not however be *Ft*RelAs natural multimeric state and might relate in the present study to the high protein concentration. Further analysis of *Ft*RelA dimeric state over a range of concentrations may elucidate the enzymes multimeric state at concentrations closer to those found *in vivo*.

Previous work on synthetase activity in long RSH enzymes from *E. coli* [30], *Mycobacterium tuberculosis* [44] and *Bacillus subtilis* [31] has demonstrated a preference for either GDP or GTP as a substrate but invariably both are accepted. Data presented in the present paper details the first example of a RelA enzyme which has an explicit specificity for one of these two main pyrophosphate acceptors. This specificity was also observed under activating conditions with stalled ribosomal complexes but interestingly not when *Ft*RelA was activated with the primary alcohol methanol (Supplementary Figure S3). The effect of methanol on protein conformation has been previously demonstrated to strengthen hydrogen bonds and weaken hydrophobic interactions [45]. Structural alterations to the synthetase-active site by methanol could therefore account for the acceptance of GDP as a pyrophosphate acceptor under these conditions and suggests the EXSD motif contributes to substrate specificity along with other structural elements of the synthetase domain. The potential importance of the structural transition in a putative catalytic loop within the synthetase domain of bi-functional enzymes with an RXKD motif has previously been reported [22]. It is therefore tempting to speculate that the EXSD motif contributes to the observed substrate specificity, although mutational analysis would be required to provide evidence for this. With equal Mg^2+^ concentrations across all activity assays, the acceptance of GDP in the presence of methanol leads us to suggest that the magnesium concentration is unlikely to be the dominant factor in determining this specificity. Further work will be required to elucidate the functional significance of this catalytic loop in RelA enzymes with the unusual EXSD motif.

Kinetic analysis of the *Ec*RelA in the absence of full activation has shown typical Michaelis–Menten kinetics [22,30]. In the present study, we demonstrate the kinetic profile for the *Ft*RelA in the absence of activating factors fits a sigmoidal curve and yields the derived kinetic parameters (termed *V*_max_ and *K_1/2_*). Measurement of both the nt products AMP and the (p)ppGpp by HPLC analysis gave comparable sigmoidal fits and calculated kinetic parameters. The observed sigmoidal curve was verified by a second technique, [^31^P]-NMR spectroscopy, which yielded comparable kinetic parameters when calculated using AMP production for rate determination. The global fit of data gave a sigmoidal curve with an *R^2^* value of 0.93 and 0.88 for ATP and GTP respectively (with a worse fit resulting from a hyperbolic curve, *R^2^*=0.85). A sigmoidal curve has been observed for the bi-functional long RSH enzyme, Rel_Mtb_, synthetase activity in the absence of activating factors [22]. This kinetic profile was linked to the RXKD motif found in the synthetase active site [22].

The substrate specificity and co-operative kinetic effects during (p)ppGpp synthesis by *Ft*RelA more closely resembles that of bi-functional RSH enzymes rather than that of other RelA enzymes. Collectively, these data demonstrate a divergence from the current classification system used for RelA enzymes [4,22] and may indicate that *Ft*RelA is instead an example of a new distinct sub-class of RelA enzymes within the protein superfamily.

Several mechanisms can account for a sigmoidal velocity curve, including the interesting possibility of allosteric regulation [46]. In 2012, Shyp et al. [14] suggested ppGpp was responsible for positive feedback regulation of *E. coli* RelA by allosteric activation. ACT domains have been shown to regulate enzyme catalytic activity by the downstream effects of binding small molecules, namely amino acids [19]. The regulatory small molecule involved in binding the ACT domain of RelA enzymes has yet to be identified. *Ft*RelA is one of only three RelA enzymes to not contain this domain [4], therefore the observation of *Ft*RelA activation by ppGpp (Figure 6) is highly indicative that ppGpp is not the regulatory ligand for RelA enzymes ACT domains.

Experiments with *Ft*RelA demonstrated that the (p)ppGpp synthetase activity could be stimulated *in vitro* by the presence of stalled ribosomal complexes formed with ribosomes from alternative species (Figure 8), as has been observed previously for RSH enzymes [47]. This weak activation of *Ft*RelA however pales in comparison with that achieved by *Ec*RelA, with maximal activities of 701.5±30.5 picomoles AMP per picomoles RelA per minute and 2952±99.14 picomoles AMP per picomoles RelA per minute respectively (Figure 8). Conversely, strong activation of *Ec*RelA (in the range of levels previously reported [11,13]), was observed in the presence of stalled ribosomal complexes formed with either *F. philomiragia* or *E. coli* MRE600 ribosomes (Figure 8). This strongly suggests that the observed lower sensitivity of *Ft*RelA to ribosomal activation is genuine and not related to the quality of purified ribosomes or the use of heterologous systems for ribosomal stalling. Key residues identified in *Ec*RelA involved in ribosomal binding were amino acids 550–682 [18]. At only 647 amino acids in length, *Ft*RelA is missing ∼22 of these identified residues involved in *Ec*RelA ribosomal binding [18] and this could account for the weaker activation observed for *Ft*RelA by stalled ribosomal complexes. Future studies may identify the mechanistic relationship between this truncation and the observed reduced activation of *Ft*RelA.

## CONCLUSIONS

*Ft*RelA contains a variety of amino acid sequence differences when compared with a wide range of other RelA enzymes, including a truncated C-terminus and an alternative EXSD active site motif. The current model for all RelA enzymes has been based on that from *E. coli*. In the present study, we describe the similarities and differences of *Ft*RelA compared with the accepted model. Observed differences include the specificity of *Ft*RelA for the pyrophosphate acceptor GTP (except in the presence of methanol). Furthermore, the sigmoidal steady state kinetics observed for *Ft*RelA are unlike those reported for *Ec*RelA, but similar to that observed for the bi-functional RSH enzyme Rel_Mtb_. Conversely *Ft*RelA behaves similarly to *Ec*RelA in its apparent ability to dimerize and its activation by both stalled ribosomal complexes and nt ppGpp. Comparison of the degree of activation by stalled ribosomal complexes for *Ft*RelA and *Ec*RelAs suggest the *Francisella* enzyme is more weakly activated. A deeper understanding of the underlying reasons behind the observed lower activation and its value to *Francisella* species forms an interesting objective for future research.

## Abbreviations

(p)ppGpp: guanosine penta- or tetra-phosphate
ACT: aspartate kinase, chorismate mutase, TyrA domain
*Ec*RelA: *Escherichia coli* RelA
*Ft*RelA: *Francisella tularensis* RelA
IDA: iminodiacetic acid
IP RP: ion pair reverse phase
ppGpp: 5′,3′-dibisphosphate guanosine
pppGpp: 5′-triphosphate-3′-diphosphate guanosine
RelA: (p)ppGpp synthetase I
RSH: RelA/SpoT homologue
SpoT: (p)ppGpp synthetase II
TGS: threonyl-tRNA synthetase, GTPase and SpoT domain
TSB: trypticase soy broth
